# On the Hydrostatic Test, and Other Proofs of the Extra-Uterine Life of the Child

**Published:** 1829-11

**Authors:** Robert Arrowsmith

**Affiliations:** Coventry.


### *On the Hydrostatic Test, and other Proofs of the Extra-uterine Life of the Child.* By Robert Arrowsmith, M.D. Coventry.

I am induced to offer some remarks on the subjects adverted to in the title of this communication, from having observed the doubtful or contradictory opinions expressed by some members of the profession, in a case of supposed infanticide, which not long since became the subject of a coroner's inquest in London: and the doubts experienced on that occasion, as in most others of a similar nature, related to the proofs of the extra-uterine life of the child. To persons accustomed to study the nature of the anatomical and physiological evidence which we now possess on this subject, it appears somewhat extraordinary that discrepancies should still be found in medical testimony; and it is to be apprehended that the doubts and difficulties which are generally experienced are the consequences rather of imperfect preparation on the part of the witness, than of the questionable nature of the proofs with which anatomy and physiology have supplied us.

Both in a civil and criminal point of view, inquiries concerning the extra-uterine life of the child are important. With regard to the former it may be observed, that in almost every country, England and America excepted, the audible crying of the child is the great test of life relied on; but in these countries a child has been decided to have been born alive who has made certain muscular movements, without, however, uttering audible sounds. In the provisions of the English law, concerning *a tenant by the courtesy of England,* it is understood that "where a man marries a woman seised of an estate of inheritance, and has by her issue born alive, which was capable of inheriting her estate, he shall, on the death of his wife, hold the lands for his life, as tenant by the courtesy of England:" and with regard to the proofs of its being born alive, Blackstone remarks, "Some have had a notion that it must be heard to cry, but that is a mistake. Crying, indeed, is the *strongest* evidence of its being born alive, but it is not the *only* evidence;" and Coke observes, "If the child be born alive it is sufficient, though it be not heard to cry, for peradventure it may be born dumb." "Crying is a proof that a child was born alive; and so is motion, stirring, and the like."* Such proofs must be established by the testimony

* Vide Beck's Medical Jurisprudence.

of by-standers, and their existence is incapable of particular elucidation by a medical witness. But whether such proofs of life be well founded or not, physiologists alone can decide; and it is highly probable that the prevailing opinion among them will always influence the verdicts of juries. That crying or respiration is the only infallible sign of life is not physiologically true; for, as children are frequently born without any indication of life, and are oftentimes resuscitated after a considerable interval, either spontaneously or by artificial means, it is manifest that the state of such children during this interval must be one of apparent death only. And, if it be alleged that the movements which children under such circumstances occasionally exhibit show only that the muscular fibre has not yet lost its contractility, the same may be affirmed of respiration, which is essentially a muscular action. What would be the decision of a jury in a case where the *vagitus vaginalis* had occurred, and the child afterwards born dead? The hydrostatic and other tests would here concur, with the testimony of the by-standers, to demonstrate that the child had performed some of the functions of independent existence; but, according to the principle laid down, "mortuus *exitus*, non est exitus," the father could not inherit.*

But it is chiefly in connexion with criminal jurisprudence that the inquiry into the signs of the extra-uterine life of the child has been prosecuted. In concealment of pregnancy, the life of the child at birth is viewed as an aggravating circumstance, and hence the negative evidence is very important to the prisoner. And, in trials for infanticide, whilst the proof of the child having been born alive furnishes only presumptive evidence in support of the charge, satisfactory evidence of its having been stillborn will refute the charge in the face of all moral testimony. And assuredly the consideration that a system of proof can never establish guilt, but may often rescue innocence from suspicion, (as is the case with the various tests of extra-uterine life,) ought to be some recommendation of its study. I say can never establish guilt, because if the life, after birth, of a child be ever so convincingly ascertained, evi-

* Chaussier, feeling something, I presume, of the absurdity of applying such finespun distinctions to political purposes, proposed to the French minister the following article: "Est reconnu et déclaré viable, apte à jouir des privileges de la societé, l'enfant dont la tête est bien conformée, qui, au plustôt, 36 heures après sa naissance, est presenté vivant et vigoureux à l'officier de l'etat civil, qui l'inscrit aussitôt sur ses registres, avec les prénoms qu'on lui donne, et les qualités des parens et des personnes qui le lui présentent."—*Mémoire Médico-legal sur la Viabilité de l'Enfant naissant.* Paris, 1826. P. 30.

dence of violent death, of criminal negligence, or wilful ill-treatment, is required to sustain the charge of infanticide.

There are three different organs in the fœtus, which, in its transition from the condition of the fœtus in utero to that of independent existence, undergo certain changes, which changes remain after death; so that their unaltered condition constitutes a proof of death before birth; their altered condition, the death of the child during or after birth; namely, the organs concerned in respiration; the circulatory apparatus peculiar to the fœtus; the digestive apparatus, with the urinary bladder and rectum. The examination of these collective signs is called the "Docimasia Biomantica," and is divided into the Docimasia Respirationis, the Docimasia Sanguinis, Circuitus, and the Docimasia Digestionis et Excretionum.

I. The *D. Respirationis* comprehends an inquiry into the cavity of the thorax: the colour, consistence, the absolute and specific weight, and the circumference of the lungs, together with the fallacies to which they are respectively liable: namely, respiration before or during birth; inflation; the emphysema pointed out by Schmidt and Chaussier; hemorrhage; and a morbid or decomposed state of the lungs.

1. The transverse diameter of the thorax is estimated by measuring from the intercostal space of one side to that of the other, taking, of course, the widest diameter; the direct diameter, by measuring from the lower end of the sternum to the body of the opposite vertebra. The level to which the arching of the diaphragm rises, is also to be remarked. Various circumstances influence the capacity of the thorax; as, for instance, the degree of maturity of the fœtus; perfect or imperfect respiration; the plumpness of the child, &c.

In mature fœtuses, and in premature ones also which are "viable," the transverse diameter of the thorax is from two and a half to three inches; the direct diameter from two to two and a half inches; and the arching of the diaphragm rises to about the fifth rib.

In children who have breathed imperfectly, the transverse diameter is from three to four inches; the direct diameter from two to three and a half inches; and the arching of the diaphragm rises to between the fifth and sixth ribs.

In children who have breathed perfectly, the transverse diameter is from three to four and a half inches; the direct diameter from three to three and a half inches. The level of the arching of the diaphragm is at the sixth rib, or between the sixth and seventh ribs.

To this general rule there are exceptions. The thorax may have a larger diameter, from being naturally but unusually spacious; or it may be increased by various morbid processes, as by inflammation, or collections of watery fluid; by inflation of the lungs of a stillborn fœtus; by breathing before or during delivery; from emphysema, or from developement of gases by putrefaction.

It may have a smaller diameter, in consequence of malformation, or from death of the fœtus by hemorrhage; from a general tabid state of the body, or from an advanced degree of putrefaction.

2. With regard to the *colour* of the lungs, it may be stated, first, that, in fœtuses which have not breathed, they are of a dark red, sometimes inclining to the brownish red of the liver, or the bluish red of the thyroid gland; and are in general darker posteriorly, on account of the subsidence of the blood there, from the position of the body.

In stillborn children, whose lungs have been inflated, they assume anteriorly the pale red colour of the thymus gland, and partially, particularly posteriorly, the reddish brown colour of the liver, or bluish red of the thyroid gland.

In children after imperfect life, (that is, in those who, by involuntary motions, have given signs of life, without breathing,) the lungs are of a dark red colour, inclining to brown or blue, whilst here and there a few cinnabar-red spots or lines are perceptible.

In those who have breathed imperfectly, or only for a short time, the lungs, on the anterior surface, are of a pale red; on the posterior surface, dark red; whilst, in different parts of the lungs, larger and more thickly-set patches, of a cinnabar-red colour, are visible.

In children who have breathed perfectly, and lived a longer time, the lungs are of a pale red, with numerous patches and lines of cinnabar-red; posteriorly, they present a dark red colour, owing to the subsidence of the blood.

In winter particularly, but even at other periods of the year, the lungs, during inspection, will become light coloured, from the influence of the air.

3. In stillborn fœtuses, the substance of the lungs, with respect to *density*, is every where compact like liver, and does not crepitate when cut; bubbles of air cannot be made to ascend by pressure under water; nor, even by means of a magnifier, can any cells be discovered distended with air.

In dead-born children whose lungs have been inflated, in those with imperfect life, or who have made some few inspirations in the womb, and in those who have breathed imperfectly, there are generally found, in the upper lobe of the right lung, several insulated groups of cells, dilated with air. In these situations the substance of the lungs is expanded, and crepitates* under the knife; the other parts are still compact, and yield no air bubbles on pressure under water. Where the cells appear dilated, air bubbles, though fine, may be pressed out.

In children who have respired perfectly, innumerable cells, distended with air, are visible over the whole surface of the lungs, united in the form of insular groups; the substance of the lungs is expanded and spongy, and crepitates audibly; and, by pressure under water, air bubbles, with froth, readily appear.

The following exceptions to the above may be enumerated: In death from suffocation after complete respiration, the lungs are found more or less compact; after death from hemorrhage, they are found flaccid. In œdema of the lungs, or when they contain pus, they are doughy. The dropsical yield water or whitish-gray foam; those whose compactness is the result of suffocation, blood, or a thick palish-yellow fluid heavier than water.

4. Before proceeding to ascertain the *absolute weight* of the lungs, it is necessary to tie the large cardiac vessels, together with the trachea, near the origin of the ductus arteriosus; the vena ascendens, and the two pulmonary arteries. The quantity of blood which the lungs contain may be ascertained by dividing the lungs into small portions, and subjecting them to considerable pressure.

Ploucquet proposed to compare the absolute weight of the lungs with the weight of the body; but the latter has been found so variable as to render this test of no value. On the contrary, the length of children is liable to much less variation; and hence Professor Bernt proposes to compare the weight of the lungs with the length of the body. The following table exhibits the general results which he has obtained from this comparison.

* I am aware of the discovery assumed by M. Piedagnel, that the lungs never crepitate except when affected with general or partial emphysema. If such were the fact, emphysema must have been present in all the children examined by M. Bernt; a conclusion not very probable. But, conceding the accuracy of M. Piedagnel's opinion, it rather confirms than militates against the inference from the crepitation of the lungs on the present occasion, as such emphysematous state is necessarily connected with respiration, either natural or artificial.—*Edinburgh Med. and Surg. Journal*, c. 223.

*Average absolute weight of the lungs of stillborn children.*

| Length. | Males. | Females. |
|---|---|---|
| From 15 to 18 inches | ʒviiiss. | ʒviij. |
| 18 to 20 | ʒix. | ʒviiiss. |
| 20 to 22 | ʒixss. | ʒix. |

The average absolute weight of the lungs of children who have respired imperfectly, amounts to, or even exceeds by some grains:

| Length. | Males. | Females. |
|---|---|---|
| From 15 to 18 inches | ʒxiij. | ʒxij. |
| 18 to 20 | ʒxiiiss. | ʒxiij. |
| 20 to 22 | ʒxiv. | ʒxiiiss. |

The average absolute weight of the lungs of children who have breathed perfectly, amounts to, and even may surpass by some drachms:

| Length. | Males. | Females. |
|---|---|---|
| From 15 to 18 inches | ʒxv. | ʒxiv. |
| 18 to 20 | ʒxvj. | ʒxivss. |
| 20 to 22 | ʒxx. | ʒxv. |

The exact quantity of blood contained in the lungs cannot be ascertained when it is coagulated. Nevertheless, if the quantity collected does not amount to one drachm, death from hemorrhage may be confidently inferred; and, if it exceed one ounce, congestion may be as safely presumed. If it be watery, we are justified in inferring a dropsical condition of these viscera.

The absolute weight of the lungs may be increased by malformations; by congestion from suffocation or inflammation, or by various other morbid conditions. It may be diminished also by malformation; by anæmia; by hemorrhage from the umbilical cord, or from wounds; by the decomposition of the blood, and its consequent escape by the trachea, &c., or by exudation.

5. With respect to the *specific weight* of the lungs of stillborn children, experiments have established that the whole lungs, and every portion of them, immediately and rapidly sink in water; that the slower sinking indicates a previous feeble effort to breathe, or an unsuccessful attempt to inflate such lungs; or a slight degree of emphysema or putrefaction. A higher degree of putrefaction or emphysema, however, or a successful inflation, will cause them to float; but under such circumstances the colour, the absolute weight of the lungs, the condition of the other viscera, and of the body generally, resolve the difficulty; particularly in conjunction with the circumstance that, in the event of emphysema or putrefaction, the portions of lung, by means of pressure, are rendered incapable of floating.

Observation has shown, with relation to the specific gravity of the lungs of those children who have breathed imperfectly, that, varying with the degree of such imperfect respiration, either the whole or some portion of the lungs (chiefly the upper lobe of the right lung, or some portion of it,) will float. The fallacies recited in the preceding paragraph may intervene here, and require the corrections before named.

The lungs of those children who have respired perfectly float either with or without the heart, altogether or in pieces, and after having been subjected to the strongest pressure. Such a capability of floating may be produced also by the several causes previously enumerated, and are to be obviated by the same means of correction.

6. *Circumference.* The lungs of stillborn fœtuses (both of which, with their rounded surface, lie in contact with the pleura, the right having commonly a greater circumference than the left,) occupy the posterior parts only of the thorax, their anterior borders extending to the pericardium. With their concave surface they cover the posterior half merely of the arch of the diaphragm; the edges are sharp, and the termination of the right middle and left upper lobe form small, ligulate* elongations.

The lungs of new-born children who have respired imperfectly, or the circumference of which has been increased by inflation, occupy, more or less, the sides of the cavity of the thorax: their anterior borders partially cover the sides of the pericardium, and their under concave surface the arch of the diaphragm, in a great measure; their borders, and the ligulate elongations of the right middle and left upper lobes, are partially or every where more obtuse.

The lungs of children who have respired perfectly, fully occupy the lateral concavities of the chest; their anterior borders cover the pericardium laterally, and their under concave surface the whole arch of the diaphragm; their borders are every where rounded, and the ligulate elongations of the right middle and left upper lobes are shorter and more obtuse.

II. *Docimasia Sanguinis Circuitus.* In considering the proofs of extra-uterine life derived from the circulatory apparatus, I shall confine myself to an examination of the

* "Zungenförmig," literally tongue-shaped.

changes experienced by that part of it immediately connected with the heart and large vessels. It will be previously requisite to describe the form and relations of the arterial canal.

The arterial canal (ductus arteriosus botalli,) originates from that part of the trunk of the pulmonary artery where it divides into its two great branches, and running parallel with the arch of the aorta and in contact with it, joins it at a very acute angle. It is about an inch long, of a cylindrical form, with a diameter equal to that of the trunk, and more than double the diameter of the two branches of the pulmonary artery; which latter are equal in thickness to a crowquill.*

1. If the fœtus have breathed for a few moments only, the aperture of the arterial canal, by which it opens into the aorta is contracted, so that the vessel assumes the form of a cone, with its base towards the pulmonary artery.

2. If it have lived several hours, or a day, the arterial canal becomes again cylindrical, but is diminished in length and thickness, equalling in the latter respect a goosequill; and consequently has a much smaller diameter than that of the trunk of the pulmonary artery, but about equal to the diameter of the two branches; which latter are now become enlarged.

3. If the child have lived several days or a week, the arterial canal is reduced in length to a few lines, and its thickness to that of a crowquill; whilst the diameter of the two pulmonary arteries is increased to that of a goosequill.†

4. It becomes entirely closed after an uncertain period: M. Billard says, most frequently after ten days.

But there are occasional exceptions to these general rules. Sometimes the contraction of the arterial canal after breathing is not at its termination towards the aorta, but towards the pulmonary artery; sometimes, although the child has

* Professor Bernt says that the fœtal circulatory apparatus is faithfully delineated in the treatise of Kilian, ("Abhandlung über den Kreislauf des Blutes im Kinde, welches noch nicht geathmet hat." Karlsruhe, 1826; 4to. mit 10 lithogr. tafeln;) and adds, that the changes effected in these vessels after breathing, which he was the first to discover, are confirmed by the examinations of Kilian.

† The conclusions above detailed were drawn by Professor Bernt from a series of Observations and Experiments, which were published in detail in 1823, (*Experimentorum Docim. Pulm. Hydrost. illustrantium Centuria I.* curante Jos. Bernt, m.d. &c. Viennæ, 1823;) and in the third edition of his Manual of Medical Jurisprudence, published at the same place in 1828, and which is my authority for most of the facts contained in this paper, he adheres to the conclusions he originally formed, and has added the confirmation of Kilian. (*Systematisches Handbuch der gerichtlichen Arzneykunde,* von J. Bernt, &c. 8vo. Wien, 1828; 3te. Auflage.)

breathed feebly, and for a short time only, the arterial canal is of the thickness of a crowquill, and therefore (as in those who have lived several days) is by far smaller than the trunk of the pulmonary artery or aorta.

III. The *Docimasia Digestionis et Excretionum* comprehends the condition of the liver, the stomach, intestines, and urinary bladder.

The liver, as a viscus whose changes with reference to respiration are important and deserving of attention, has been but little examined. Dr. Beck first proposed to ascertain the weight of the liver before and after respiration, and conceived it would be a means of verifying the static test of Ploucquet. Professor Bernt says that Dr. Beck is mistaken in supposing that the principle on which his proposal rests is exposed to no greater practical objections than the test of Ploucquet. He (M. Bernt) has instituted one hundred experiments on the state of the liver, and he gives the following as the result of his inquiries:

1. The situation of the liver and length of the abdominal cavity, are relative to the situation of the diaphragm and capacity of the thorax. The arched surface of the diaphragm in the fœtus protrudes farther into the cavity of the thorax than in newly born children. This relation may be deranged by a relaxation of the diaphragm, from putrefaction.

2. The colour of the liver varies (without reference to the child having been born living or dead,) from reddish brown to dark or blackish brown. The darkest colours are most frequently met with in premature children, or in a congested state of the liver.

3. The edges of the liver project out of the hypochondrium, both in dead and living born children. Its absolute weight in the former (the length of the body being from fifteen to twenty-two inches) was from ℥iiiss. to ℥viiss.; in those which had breathed imperfectly, from ℥iiss. to ℥vij.; and in such as had breathed perfectly, from ℥iiss. to ℥viiiss.

4. The blood contained was quite fluid or semifluid, for the most part dark red, but sometimes, in dead-born children, light red. The gallbladder was pyriform in those only who had lived several days, and for the most part contained some reddish yellow bile.

5. The ductus arantii was always open during the first days after delivery: later, it becomes contracted, and is generally found closed after the sixth day.

It is almost superfluous to add any thing relative to the condition of the other abdominal organs. The changes occurring in them are either so slow or so obvious as to require no indication.

It has been well observed by several authors, that, in inquiries into the proofs of extra-uterine life, as in medico-legal investigations of every kind, it should never be forgotten that each proof, considered in itself, and without connexion with others, is necessarily unsatisfactory. But, in proportion as proofs and probabilities are multiplied will fallacy and error be diminished; and if the various proofs mutually support each other, they supply a body of evidence so strong and luminous as to satisfy both the physiologist and the jurist.

It will not be deemed unimportant, perhaps, to subjoin the following compendious summary:

*a.* The fœtus may be concluded to have died before birth, and never to have breathed,

1. If the transverse diameter of the thorax be from two to three inches, the direct diameter from two to two and a half inches, and the arching of the diaphragm extend to the level of the fifth rib.

2. If the colour of the lungs be dark red, or inclining to the brownish colour of the liver, or the bluish colour of the thyroid gland.

3. If the substance of the lungs have the compactness of the liver; if there be no cells distended with air on their surface; if they do not crepitate when cut; and if they yield no bubbles of air when submitted to pressure under water.

4. If the absolute weight of the lungs, compared with the length of the body, corresponds with the ratio already pointed out in the table (p. 417,) as the average ratio in dead-born children.

5. If the lungs in connexion with the heart, or without it, or every portion of each lung, when divided, rapidly sink in water; and if they are respectively heavier than water by several grains or scruples.

6. If the lungs occupy the posterior part of the cavity of the thorax, extending by their anterior edges merely to the pericardium; and with their under concave surface covering only the posterior half of the arch of the diaphragm; if the borders are thin, and the ends of the right middle and left upper lobes form small, pointed, ligulate elongations.

7. If the umbilical vessels, the ductus arantii, and the other circulatory apparatus peculiar to the fœtus, be pervious; and if the arterial canal have a cylindrical form, with a diameter equal to that of the aorta, and nearly three times greater than that of the two pulmonary arteries.

*b.* It may be concluded that the child has lived a short time after birth, and has breathed imperfectly,

1. When the transverse diameter of the thorax is from three to four inches, the direct diameter from two to three and a quarter inches; and when the level of the arch of the diaphragm is between the fifth and sixth ribs.

2. When the lungs are of a dark red colour, sometimes inclining to brown or blue; whilst at the same time they exhibit, in some part or other of the lungs, but particularly in the edges, bright or cinnabar-red spots or stripes.

3. When cells distended with air are visible in the surface of the lungs, (particularly in the upper lobe and edges of the right lung,) collected in insulated groups, and hence immediately connected with compact portions of the substance of the lung; hence also crepitating or not, according to the portion which is cut, and yielding bubbles of air, from pressure under water of the expanded portions.

4. When the absolute weight of the lungs is manifestly increased, in comparison with the length of the body. (Vide p. 417.)

5. When the whole lungs, with or without the heart, sink in water, at the same time that one or other lobe, or several or a few portions, float.

6. When the lungs occupy more or less the lateral parts of the cavity of the thorax; when their anterior edges and the ligulate elongations of the right middle and left upper lobes, are partially or every where become obtuse.

7. If the umbilical vessels, the ductus arantii, and the other circulatory apparatus peculiar to the fœtus, be yet pervious; the arterial canal having, however, a diminished thickness, namely, that of a goosequill, and consequently a far less diameter than that of the trunk, but equal to the now increased diameter of the two branches of the pulmonary artery.

*c.* It may be concluded that the child has lived a longer time after birth, and has breathed perfectly,

1. When the transverse diameter of the thorax is from there to four and a half inches; the direct diameter from three to three and a half; and the level of the arching of the diaphragm is between the sixth and seventh ribs.

2. If the colour of the lungs be generally pale, with numerous cinnabar-red spots, stripes, and edges; and are merely dark red on their posterior surface, on account of the subsidence of the blood from its own gravity.

3. If innumerable cells distended with air, and collected into insular groups, be plainly visible on the surface; if the substance of the lungs be every where expanded and spongy, crepitating audibly when cut, and yielding, on pressure under water, numerous air bubbles, or a reddish froth.

4. When the absolute weight of the lungs, in comparison with the length of the body, is manifestly and considerably increased. (Vide Table, p. 417.)

5. If the lungs, in connexion with the heart, and still more without it; if each lobe separately, and all the portions of it, when divided, project above the surface of the water, and float even after the strongest pressure, and are by far respectively lighter than water.

6. When the lungs quite fill the lateral parts of the cavity of the thorax, their anterior edges cover the sides of the pericardium, and their under concave surface the whole arch of the diaphragm. When their edges are every where rounded, and the ligulate elongations of the right middle and left upper lobes are shorter and obtuse.

7. When the length of the arterial canal is contracted to some lines, its thickness to that of a crowquill; whilst the thickness of the two pulmonary arteries is equal to that of a goosequill.

8. When the stomach occupies a completely transverse position, and is either freed from the albuminous fluid which in the fœtal state it contains, or presents traces of milk, or other extraneous matters. When the bowels are in some degree, or altogether, freed from meconium, and instead of it contain yellowish fæces; and when the urinary bladder is empty.

It appears to me necessary to add a few words with respect to *vaginal inspiration*.

If it be true that a child may inspire whilst in the vagina, or after the protrusion of the head, and yet die before the complete expulsion of the body is effected, a source of fallacy exists, which requires a mode of correction not adverted to in the preceding observations.* Professor Marc has argued that vaginal inspiration is impossible, from the incapability of expanding the chest, whilst the child is retained in the maternal passages. The testimony, however, of Schmidt, Osiander, Capuron, and others, leaves no doubt of the fact; and the unique case related, in the ad-

* We are certain, from our own experience and observation, that a child may inspire *after* the protrusion of the head, and yet die before the complete expulsion of the body.—Editors.

mirable work of Dr. Beck, by Dr. Hosack, has confirmed it, and established the possibility of death afterwards, before complete expulsion. In the relation of the case, Dr. Hosack recorded his belief that this child could not have been delivered without assistance; and thus sanctioned the general opinion of medical jurists, that vaginal respiration does not occur under circumstances which can create doubt, because it only takes place either where the delivery is so promptly effected as to be without difficulty or danger to the mother or child, or in presentations which render assistance indispensable; and this opinion is supported by all present experience. Presentations of the face or the feet are the cases in which vaginal respiration is most likely to occur.

In face presentations, my own experience enables me to say that a child may respire before birth; and although delivery in such presentations is exceedingly painful and difficult, and most frequently demands professional aid, we cannot say that unassisted delivery is impossible. (Vide Baudelocque, Heath's translation, vol. ii. p. 224.) In such cases the turgid state of the face, and other marks, so plainly indicate the nature of the presentation as to preclude mistake.

In footling cases, the other kind of presentation in which vaginal inspiration may occur before birth, and yet the child die before delivery, I apprehend little difficulty would be felt by any moderately judicious and experienced person in explaining the case. The absence of the usual tumor at the vertex would demonstrate the nature of the presentation; and, if any marks of injury existed on the child, their nature, situation, and direction would show whether they were the natural consequences of the assistance which a woman might have rendered herself. Foderé (*Traité de Médecine Légale,* iv. 500,) has detailed an instance of this nature; and his report on it is so judicious and full of instruction, as to furnish very valuable assistance in similar cases. Hence it appears that the possibility of vaginal inspiration does not invalidate the before-mentioned proofs of extra-uterine life, since the circumstances under which it may happen are known, and can be properly appreciated.

*Coventry; Sept.* 8*th,* 1829.

